# Supplementary material for: Splice-Junction-Based Mapping of Alternative Isoforms in the Human Proteome
Source: Cell Rep. Author manuscript; Available in PMC 2020 Jan 15. (PMC6961840; doi:10.1016/j.celrep.2019.11.026)
Supplement: 2 [file NIHMS1546469-supplement-2.zip › DF1/PXD006675/PulmonaryVein/PulmonaryVein_24_MAP4_DSYVPLELAK.pdf]

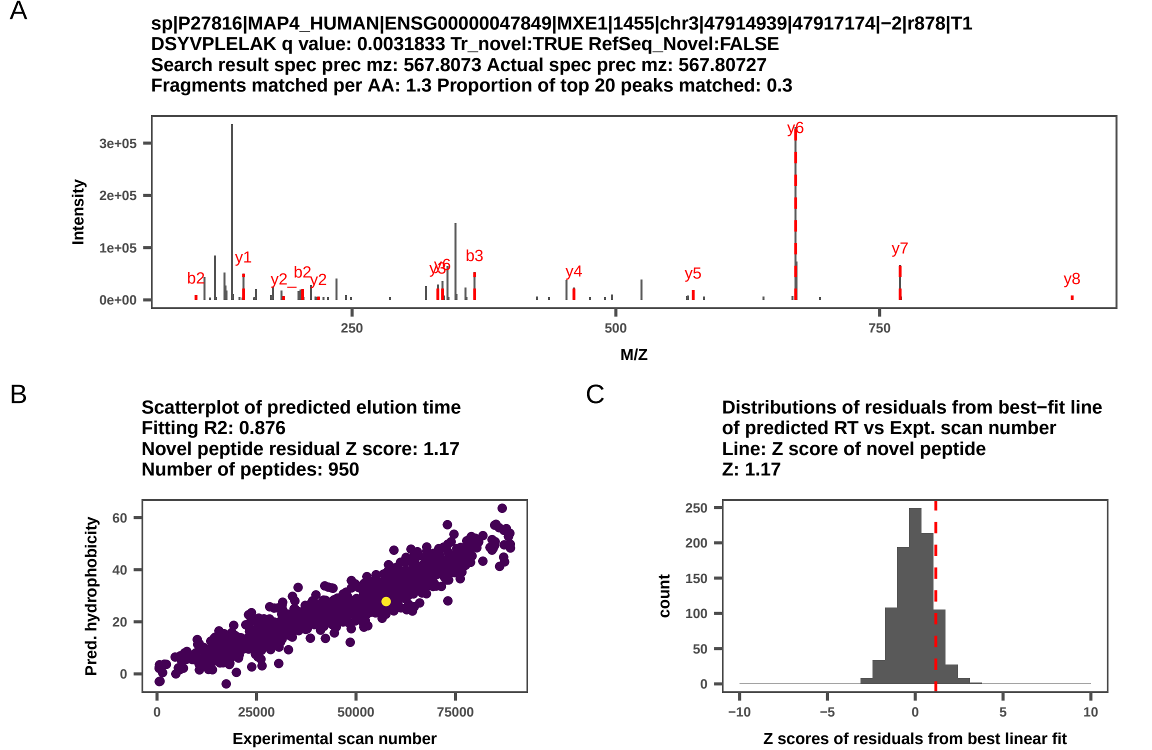

A
sp|P27816|MAP4_HUMAN|ENSG00000047849|MXE1|1455|chr3|47914939|47917174|−2|r878|T1
DSYVPLELAK q value: 0.0031833 Tr_novel:TRUE RefSeq_Novel:FALSE
Search result spec prec mz: 567.8073 Actual spec prec mz: 567.80727
Fragments matched per AA: 1.3 Proportion of top 20 peaks matched: 0.3
Intensity
3e+05
2e+05
1e+05
0e+00
b2
y1
y2_
b2
y2
y3
y6
b3
y4
y5
y6
y7
y8
250
500
750
M/Z
B
Scatterplot of predicted elution time
Fitting R2: 0.876
Novel peptide residual Z score: 1.17
Number of peptides: 950
Pred. hydrophobicity
60
40
20
0
0
25000
50000
75000
Experimental scan number
C
Distributions of residuals from best−fit line
of predicted RT vs Expt. scan number
Line: Z score of novel peptide
Z: 1.17
count
250
200
150
100
50
0
−10
−5
0
5
10
Z scores of residuals from best linear fit
